# Supplementary material for: Using mass spectrometry to investigate fluorescent compounds in squirrel fur
Source: PLoS One. 2022 Feb 22;17(2):e0257156. doi: 10.1371/journal.pone.0257156 (PMC8863215; doi:10.1371/journal.pone.0257156)
Supplement: S1 File — The fluorescence analysis of all fur samples observed at an excitation length of 400 to 600 nm, excited at a fixed emissions peak of 350 nm. (PDF) [file pone.0257156.s001.pdf]

Fluorescence analysis of fur samples:

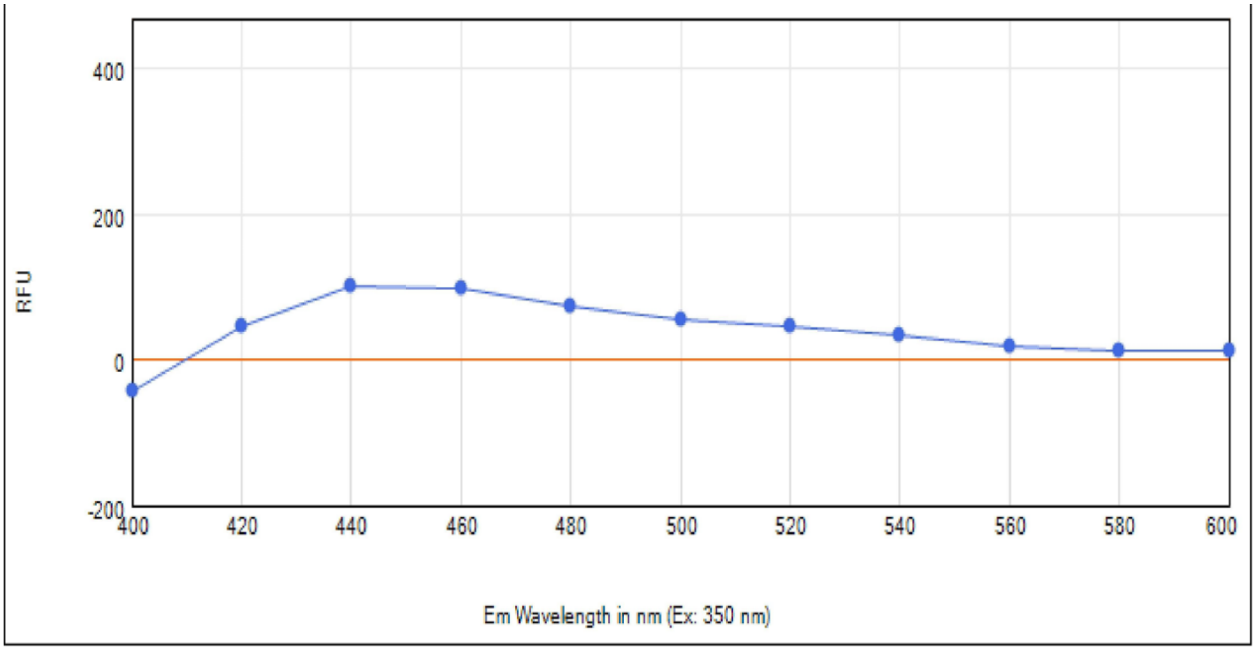

Supplementary Figure 1: The fluorescence analysis of the dorsal fur of the northern flying squirrel at an excitation wavelength of 350 nm with emissions at 400 nm to 600 nm.

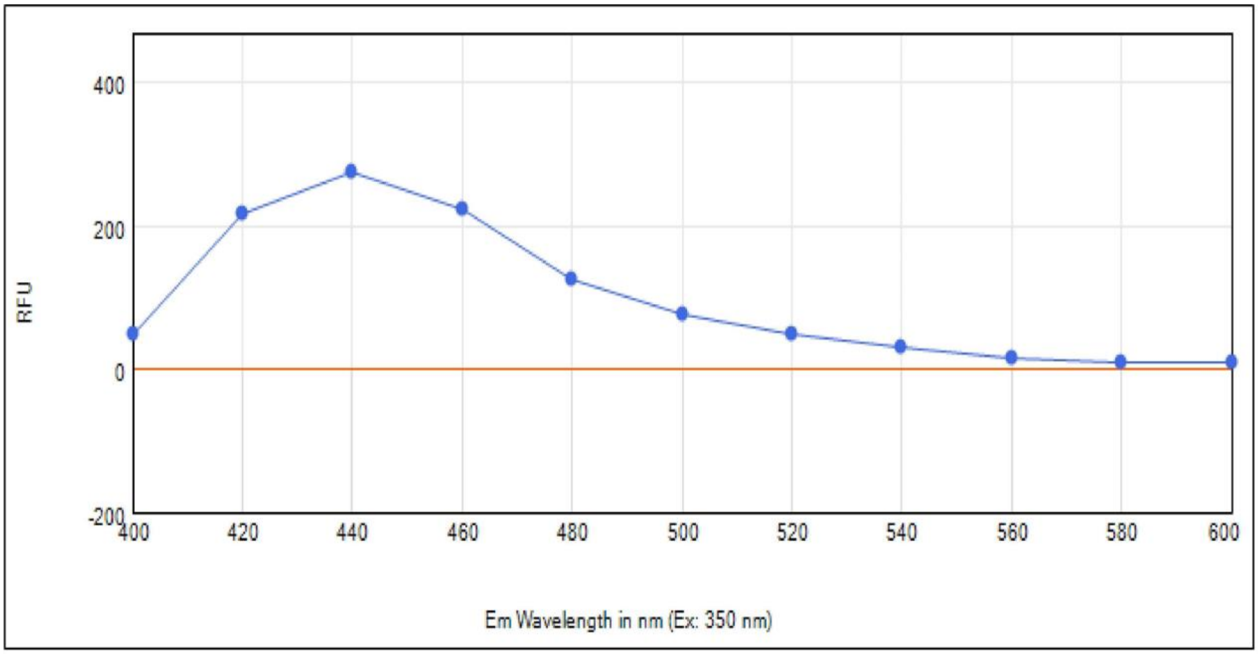

Supplementary Figure 2: The fluorescence analysis of the ventral fur of the northern flying squirrel at an excitation wavelength of 350 nm with emissions at 400 nm to 600 nm.

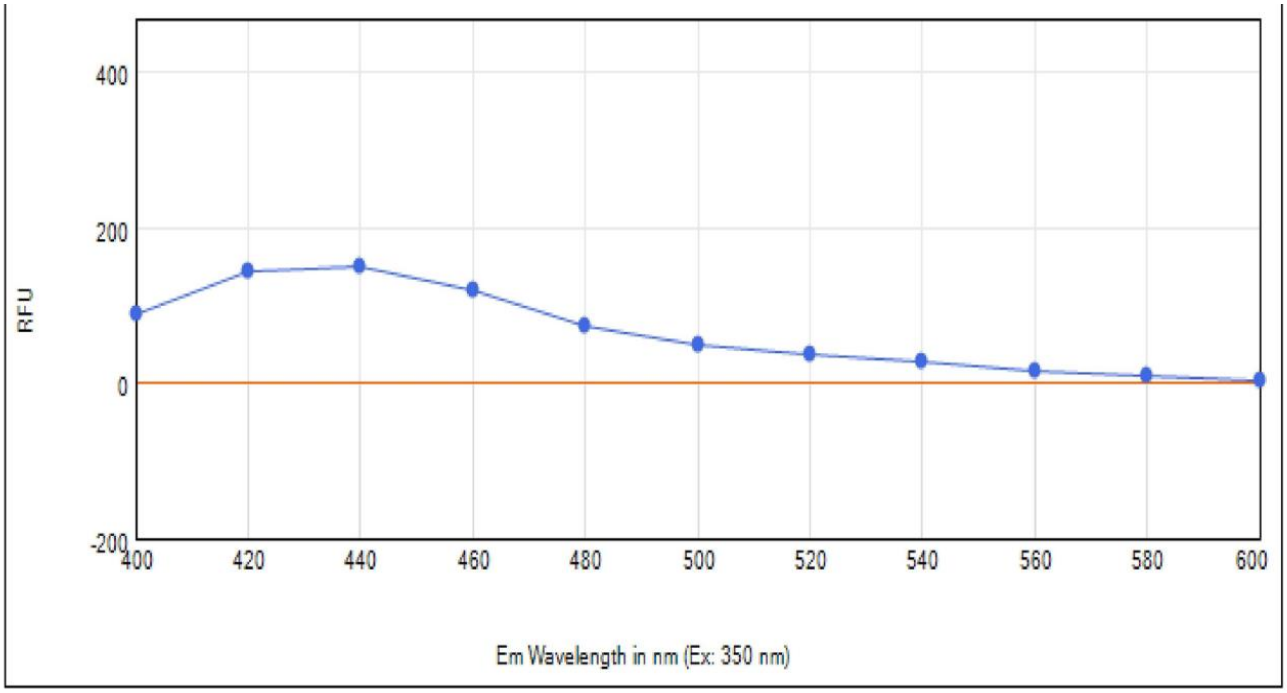

Supplementary Figure 3: The fluorescence analysis of red squirrel fur at 350 nm showing an emission wavelength of 400 nm to 600 nm.

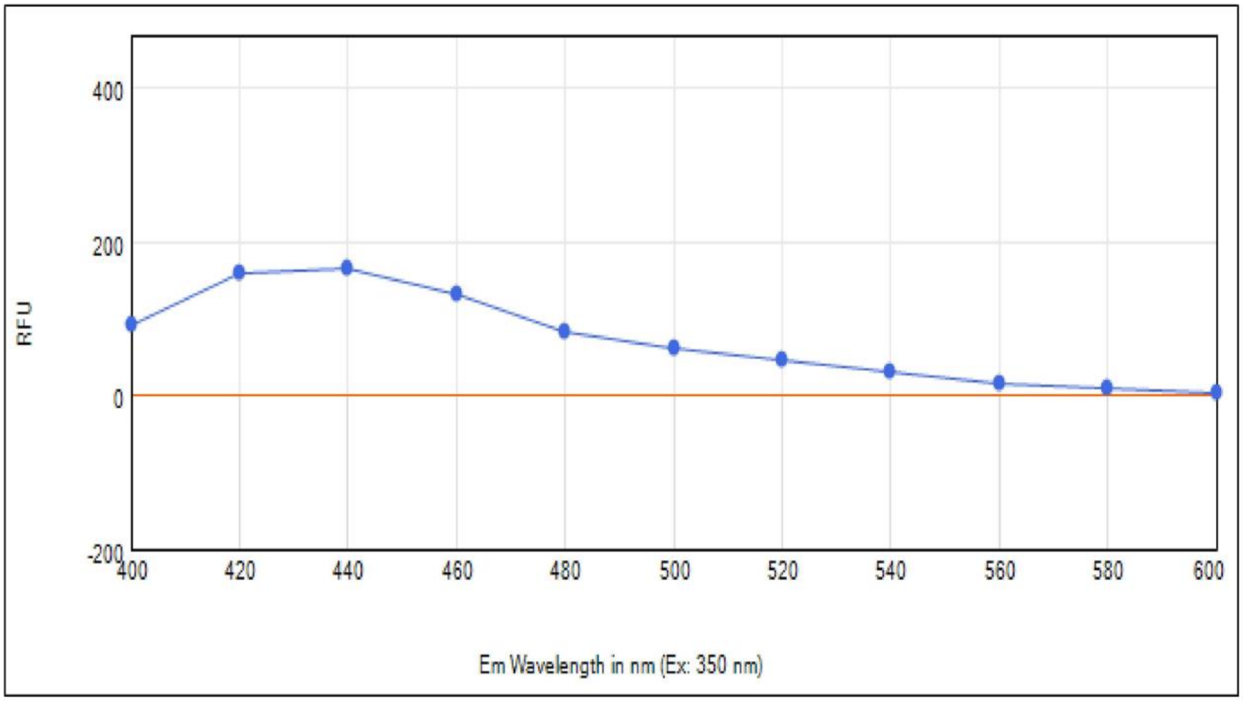

Supplementary Figure 4: The fluorescence analysis of gray squirrel fur at an excitation wavelength of 350 nm showing an emission spectrum of 400 nm to 600 nm.

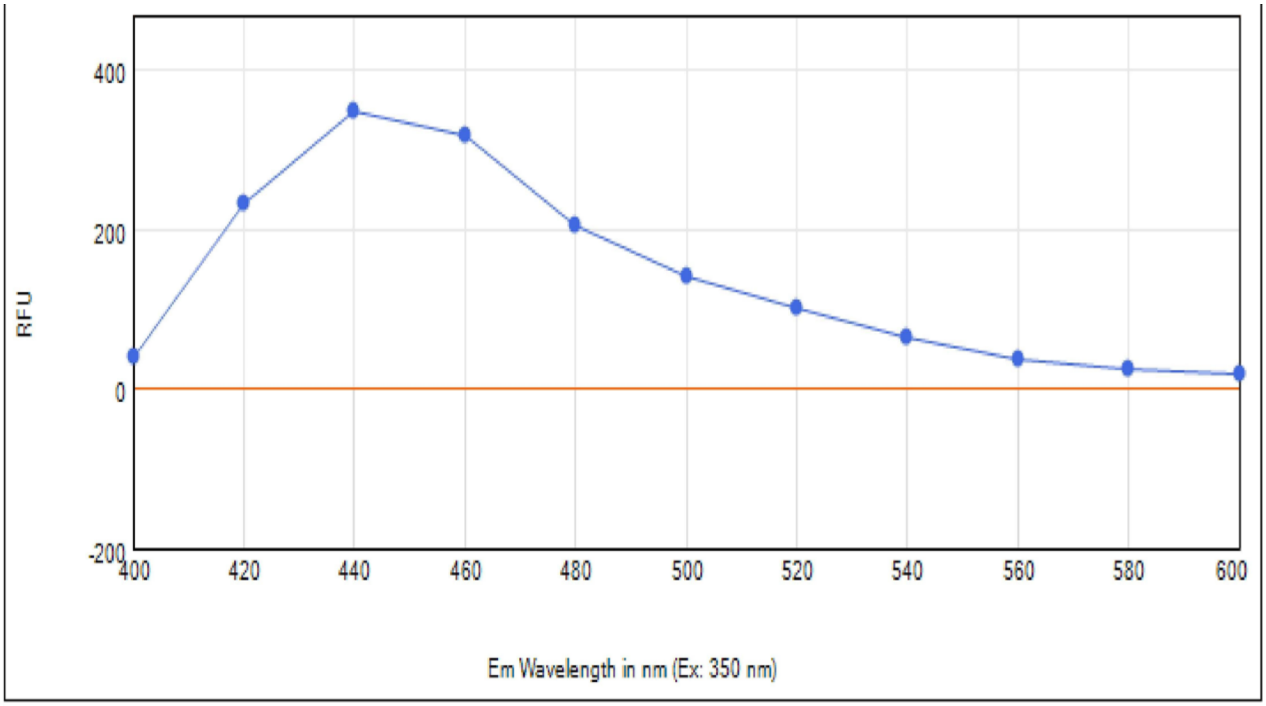

Supplementary Figure 5: The fluorescence analysis of dorsal fur from the southern flying squirrel, excited at a wavelength of 350 nm showing possible emissions from 350 nm to 600 nm.

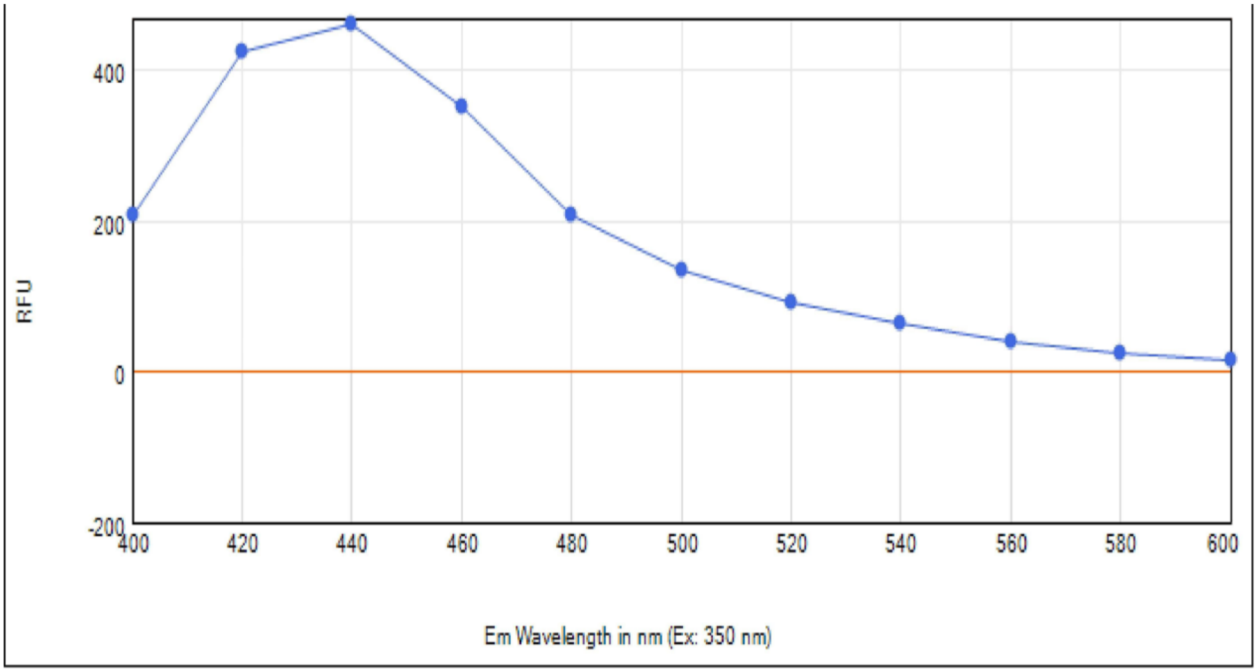

Supplementary Figure 6: The fluorescence analysis of ventral fur from the southern flying squirrel, excited at a wavelength of 350 nm with fluorescence observed at a range of 350 nm to 600 nm.

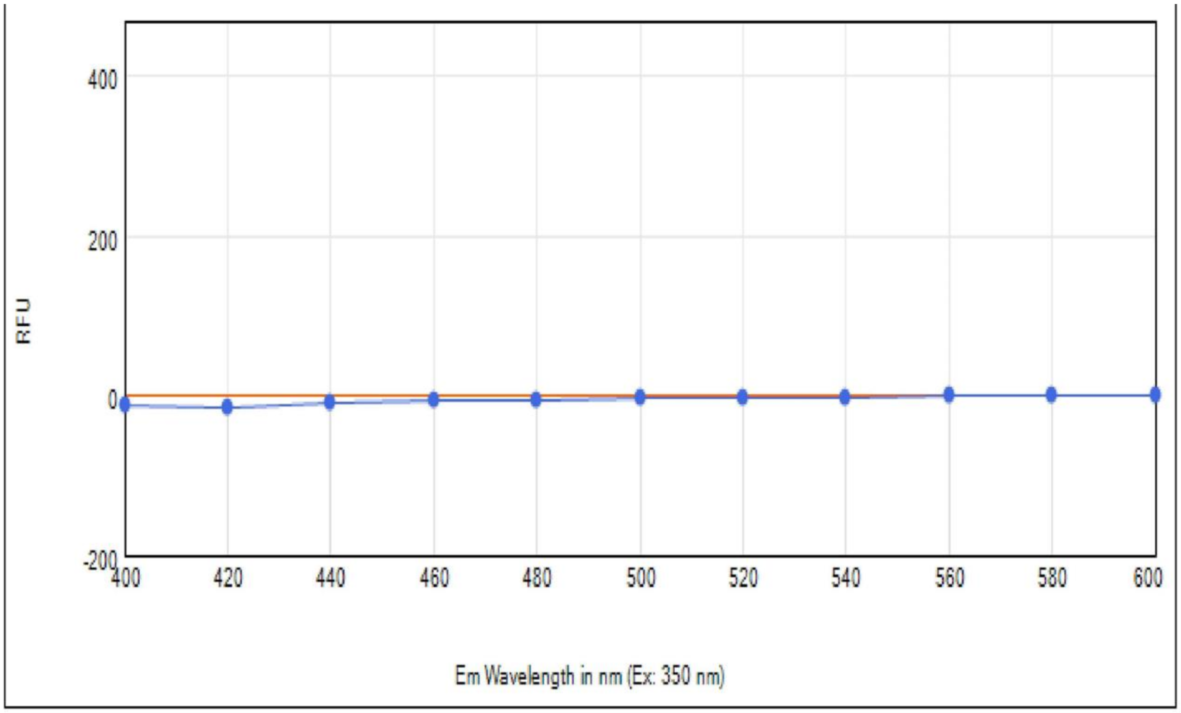

Supplementary Figure 7: The fluorescence analysis of our control sample, containing only methanol labelled as Blank 1, showing a sample excited at 350 nm observing possible fluorescence at a range of 400 nm to 600 nm.

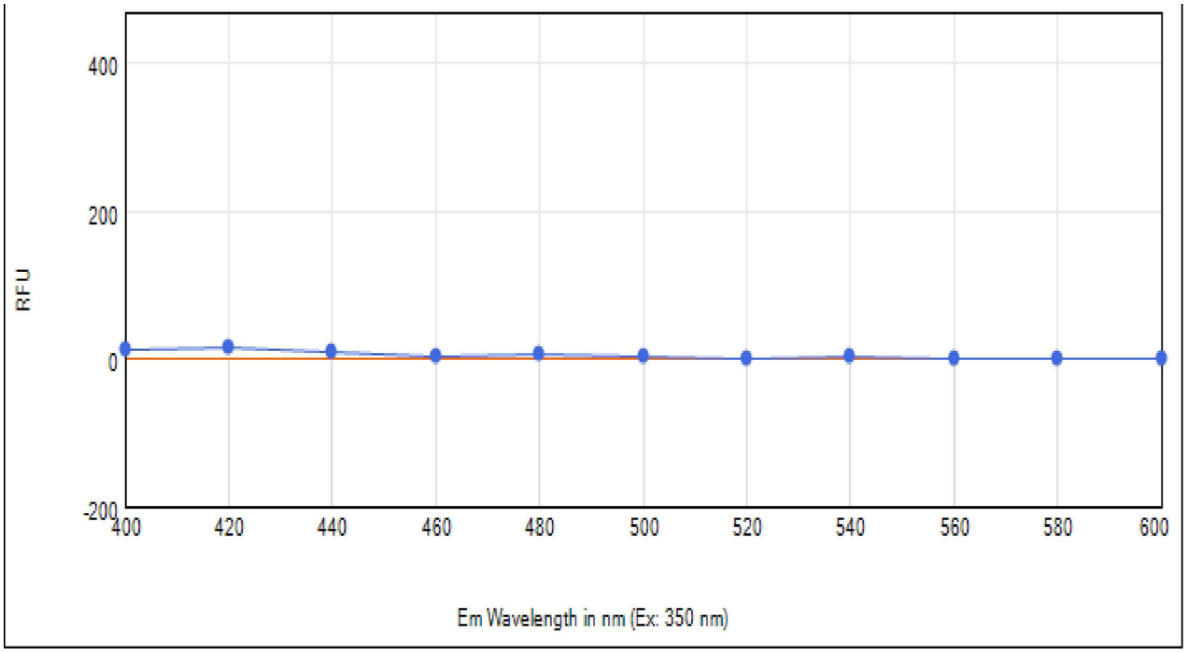

Supplementary Figure 8: The fluorescence analysis of our control sample, labeled as blank 3, excited at a wavelength of 350 nm and observed for possible fluorescence at an emissions range of 400 nm to 600 nm.
