# Supplementary material for: Using mass spectrometry to investigate fluorescent compounds in squirrel fur
Source: PLoS One. 2022 Feb 22;17(2):e0257156. doi: 10.1371/journal.pone.0257156 (PMC8863215; doi:10.1371/journal.pone.0257156)
Supplement: Attachment — Submitted filename: Response to Reviewers.docx [file pone.0257156.s005.docx]

**Editor comments:**

1. Please ensure that your manuscript meets PLOS ONE's style requirements, including those for file naming.

**R1.** **Changes have been made to the format of headings/sub-headings and files have been re-named in accordance with PLOS one journal requirements.**

2. In your Methods section, please provide additional details regarding the animals used in your study and ensure you have described the source. For more information regarding PLOS' policy on materials sharing and reporting

**R2.** **No live animals were used in this study. Additional methods have been added to line 143-145 to reflect this**.

3. Thank you for stating the following financial disclosure: “This study was funded by the natural sciences and engineering research council discovery grant, JB. ”Please state what role the funders took in the study.  If the funders had no role, please state: "The funders had no role in study design, data collection and analysis, decision to publish, or preparation of the manuscript." If this statement is not correct you must amend it as needed. Please include this amended Role of Funder statement in your cover letter; we will change the online submission form on your behalf.

**R3. We have added a statement that the funders had no role in the study to the cover letter.**

4. Thank you for stating the following in the Acknowledgments Section of your manuscript: “The authors are grateful to the Canadian Foundation for Innovation, and the Ontario Research Fund for funding instrumentation, including the Bruker SolariX XR FT-ICR mass spectrometer, in the Water Quality Centre of Trent University” We note that you have provided additional information within the Acknowledgements Section that is not currently declared in your Funding Statement. Please note that funding information should not appear in the Acknowledgments section or other areas of your manuscript. We will only publish funding information present in the Funding Statement section of the online submission form. Please remove any funding-related text from the manuscript and let us know how you would like to update your Funding Statement. Currently, your Funding Statement reads as follows: “This study was funded by the natural sciences and engineering research council discovery grant, JB.” Please include your amended statements within your cover letter; we will change the online submission form on your behalf.

**R4. Changes have been made to the acknowledgments section to remove reference to funding.**

5. Please ensure that you refer to Figure 1 in your text as, if accepted, production will need this reference to link the reader to the figure.

**R5. The reference to Fig. 1 was accidently removed in an earlier version of the manuscript and has now been re-added to line 134. Fig. 1 now has an added figure title and caption following the paragraph it is first mentioned in.**

6. Please include captions for your Supporting Information files at the end of your manuscript, and update any in-text citations to match accordingly. Please see our Supporting Information guidelines for more information

**R6**. **Supplementary file titles and captions have been added.**

**Reviewer 1:**

7. As a general note, manuscripts are typically written with line numbers present so it is easier for reviewers to give feedback.

**R7. Line numbers have been added on this version of the manuscript to aid with continued revision.**

8. Page 1, Abstract “Some factor currently unexplained...” This sentence is unclear.

**R8. This sentence has been revised, see lines 24-25.**

9. Page 2, Paragraph 2 “As the electron transitions...” The paper says longer wavelength of light, but it does not state what it is longer than. The photon that is emitted is of lower energy (i.e. longer wavelength) that the original excitation photon, but this is not clear as written in the text. Since this is a biology paper, rather than get into nuances about this point, one could simply say “As the electron transitions back to its ground state, excess energy is lost in the form of a photon.”

**R9. We have added the reviewer’s suggested wording on Lines 51-52.**

10. Page 2, Paragraph 2 “Since fluorescence is not...” Replace “non-energized state” with “ground state.”

**R10. We have changed “non-energized state” to “ground state” on line 54.**

11. Page 2, Paragraph 2 “Since fluorescence is not...” The phrase “transmit fluorescence wavelengths” is very awkward and formally incorrect. One would just say “fluoresce.”

**R11. We have changed lines 54-55 to now say fluoresce instead of “transmit fluorescent wavelength”.**

12. Page 2-3, Paragraph 2 “Other molecules that contain...” Replace “different” with “various.”

**R12. Line 58 now says “various” instead of “different.**

13. Page 3, Paragraph 1 “The fluorescence of aromatics...” This is incorrect. The LUMO is higher in energy than the HOMO, therefore you cannot have a deexcitation from the HOMO to the LUMO; its LUMO to HOMO. Please correct this sentence.

**R13. This error has been corrected to now have the correct order of the HOMO-LUMO gap see Line 60-63.**

14. Page 3, Paragraph 1 “Aromatic rings with highly...” The sentence is structured such that it implies the HOMO-LUMO gap emits UV light, which is technically incorrect. The HOMO-LUMO gap dictates – to an extent – the energy of the photon that is emitted, and the energy of the emitted photon dictates the wavelength of light we observe.

**R14. This has been revised to better reflect the process of fluorescence. See lines 62-65.**

15. Page 3, Paragraph 3 “In fish, there are...” Add the following citation: Park HB et al (2019) Bright green biofluorescence in sharks derives from Bromo-Kynurenine metabolism. iScience 19:1291–1336. <https://doi.org/10.1016/j.isci.2019.07.019>

**R15. Added citation to line 80.**

16. Page 4, Paragraph 2 “Tryptophan is one of...” Tryptophan is one of the three natural/proteinogenic aromatic amino acids. There are many aromatic amino acids.

**R16. Change made on line 113.**

17. Page 5, Paragraph 1 “The colour of fluorescence...” Fluorescence is not within a compound.

**R17. We have changed the wording here, see line 118.**

18. Page 7, Paragraph 2 “Fluorescence analysis: Aliquots ( 2 ml) Remove space before ( and 2.

**R18.** **Fixed incorrect spacing on line 182.**

19. Page 8, Results, Paragraph 1 “Several of these compounds...” What does this mean? What are the exact criteria being used to determine whether compounds have the necessary chemical structure to indicate capability to fluoresce?

**R19. Lines 55-79 we list a general set of criteria that we expect would indicate a molecule is capable of fluorescence. This is not to say that it is impossible for molecules that don’t meet these criteria to exhibit fluorescence, but rather that molecules that are known to fluoresce typically have these characteristics. These include the presence of one or more aromatic rings, or other cyclic structure, and the presence of one ore more conjugated pi-bonds.**

20. Page 9, Paragraph 4 “Our analysis of the...” The fluorescent data looks to be consistent with eumelanin. This compound is present in keratinaceous tissue and would explain why some of the non-fluorescent specimen also exhibit emission.

**R20.** **It is possible that the observed compound here is eumelanin, however it is beyond the scope of this study to identify specific fluorophores in the fur of non-fluorescent species.**

21. Page 11, Paragraph 2 “Our findings are consistent...” If the authors believe porphyrins could responsible for the fluorescence in these squirrel samples, then why not use excitation and emission wavelengths that can detect porphyrin-based fluorescence? The authors excite at 350 nm and detect emissions from 400-600 nm; however, biological porphyrins typically have absorbance maxima near 400 nm and emission maxima near 650 nm. The manner in which the authors conducted the fluorescence analysis would make it very difficult identify porphyrins as a fluorescent agent in this study

**R21. The identification of porphyrins was beyond the scope of our study. Instead, we aimed to undertake a comparison of potential compounds across a group of species using methods similar to those recommended by Kohler et al. (2019). We agree that a follow up study targeting porphyrins specifically should use different excitation and emission wavelengths.** **To clarify the implications of our findings to our hypothesis, we have added information on lines 311-317 and lines 381-382.**

22. Page 13, Paragraph 1 “It is also possible...” This is easily checkable via black-light, fluorescence spectrometry, absorbance spectrometry (if porphyrin-based), etc.

**R22. Because this is a novel use of mass spectrometry, we felt it was important to include that it is always possible for this type of error to occur.**

23. Table 3 A potential formula of CH5B2 is completely ludicrous, especially as the ion observed is [M+Cl]-

**R23. While this chemical formula does agree with the observed *m/z*, we agree with the reviewer that this is not a reasonable compound to be observed in mammal fur, and we have removed it from the table. Several chemical formulas containing fluorine, have also been edited. While these chemical formulas have a slightly less mass accuracy with the observed *m/z* values, they are more probable to be observed in mammals.**

**Reviewer 2:**

GENERAL COMMENTS

25. you find the same molecules in fluorescing and non fluorescing squirrels, and you even show that the supposedly "non fluorescing squirrels" are actually capable of some degree of fluorescence. Is it possible that these results can be explained by how much of the molecules is present in each? Maybe noticeable, measurable fluorescence is determined by amount (rather than presence or absence).

**R25. Yes, this is a plausible explanation. The earlier works of Levin and Flyger (1973) suggested that the bones of the eastern fox squirrel were fluorescent because of the accumulation of porphyrins, specifically Uroporphyrin I. Uroporphyrin I is not necessarily always found in mammals because it is an isomer of Uroporphyrin III that forms in the absence of certain enzymes that stimulate proper porphyrin synthesis in most mammals. However, all mammals do have porphyrins, while not having fluorescent bones. To highlight this as a potential explanation for fluorescence see lines 296-299 and 374-377.**

26. Fluorescence does not create light. Fluorescence is not glowing. Needless to say, YOU do not make these classic errors in this well-written paper, but the ill-equipped reader is also not directed to address these common misconceptions. This is a stylistic request of mine which you are free to ignore, but could you add some text to explain these common misconceptions?

**R26. We have added a short explanation on lines 47-49.**

MINOR COMMENTS

27. *Page 2
- missing comma after "fully understood"- give an example of aromatic molecules?
- when you say that molecules with a small HOMO-LUMOgap emits UV wavelengths, I find myself taking a pause to sort out whether in this case the molecules are fluorescing UV light-- rather than as is typically described in nature, absorbing UV light and emitting higher-wavelength light in the blue or green or even higher. Is it correct as written?*

**R27.**

A) **A comma has been added after fully understood.**
B) **An example has been added on line 56**
C) **The HOMO-LUMO gap itself does not emit UV light, rather the HOMO-LUMO gap dictates the energy of the photon that is emitted. See R12 and R13.**

28. Page 3
-*briefly explain what a conjugated pi-bonded system is
- in the third paragraph, can you briefly add what wavelengths of light are fluoresced for these examples? It is useful to know what the incident light color is and what the fluoresced light color is*

**R28.**

A) **We have added a brief sentence to generally describe what a pi-bonded system is. See lines 68-69.**
B) **The incident light colour of each of these fluorophores has been added where applicable and has been left more general for taxa that fluoresce across the entire, or almost entire spectrum. Where possible, we have also added emissions these species are observed under.**

*29. Page4
- can you say more about the evidence that nocturnal animal can detect fluorescent pigmentation? This is a conclusion that requires that there is ample UV illumination to produce fluorescence, and that the fluoresced light (as a subset of all reflected light) is sufficient to be detected by the animal in the relevant wavelength range.
- when you say fluorescent porphyrins break down upon repeated exposure to UV, so we should expect it to be more common in nocturnal mammals, can you explain with one more sentence what you mean? that is, fluorescence is just a side effect of porphyrin chemistry, and therefore only mammals with limited exposure to UV should use lots of porphyrins?*

**R29.**

A) **You are correct with this conclusion, the two studies we are aware of that specifically look at mammals’ ability to detect UV light suggest that the density of various photoreceptor proteins, and the absence of a yellow-eye lens that would filter UV light in diurnal species, may indicate that at least some nocturnal species can detect UV light in otherwise low-light conditions. To better explain these two studies, we have added information on lines 100-105.**
B) **Our current understanding would predict that all mammals would have similar levels of porphyrins (or variation in porphyrin abundance may be independent from fluorescence to a certain degree). Because porphyrins are used in the heme production pathway, fluorescence is not the primary function of these compounds. Rather, if fluorescence is largely dictated by the accumulation of porphyrins in the fur, and UV light degrades these compounds, we should see similar levels of porphyrins in the fur of any mammal regardless of active period. However, porphyrins within diurnal mammals should degrade and only produce low, or no measurable level of fluorescence, versus nocturnal mammals that should be fluorescent. This hypothesis, presented by Toussaint et al. (2021) would suggest that all, or most, nocturnal mammals should be fluorescent. See line 106-110.**

*30. Page 5
- the paragraph starting "fluorescence in eutherian" is an excellent paragraph that frames the problem and question really well. Perhaps this paragraph should appear at the start of the introduction or near there? I leave it up to your discretion.*

**R30. We prefer to leave this as is.**

*31. Page 6
- is there a table of specimen numbers that you can reference here?
- accidental comma instead of period in G. volans
- how do you know the non-fluorescing species are non-fluorescing?- did you take the fur from the same "spot" on each animal within the dorsal and ventral pelages (in case of variation)?*

**R31.**

A) **We have added a table with a list of all specimens examined with the appropriate number of ventral/dorsal samples taken, with the category (fluorescent fur vs not) line 152.**
B) **Removed comma and added a period.**
C) **All fur samples were collected from specimens that were observed to be either fluorescent (flying squirrels) or non-fluorescent (chipmunks, gray squirrels and red squirrels) under a 395nm blacklight table caption 1, lines 152.**

*32. Page 7
-why only scan for emissions between 400 and 600 nm?*

**R32.** **All fluorescent samples within this study, and the original paper by Kohler et al. (2019) are excited by a blacklight at 395nm. Because the ventral portion of the flying squirrel emits a pink colour, and the dorsal portion of the flying squirrel emits a blue colour, we predict the emissions range to be somewhere between 400 and 600 nm. A justification for our emissions selection has been added to the methods here 188-190:**

*33. Page 9
- Is it possible that the compounds were present in very different levels in the different species, accounting for observable versus non observable fluorescence?
- indeed as I continue reading it appears that the gray and red squirrel fur are indeed capable of a small amount of fluorescence*

**R33.**

A) **Yes, measuring the abundance or accumulation of specific potential fluorophores is a bit beyond the scope of this study.**
B) **Currently, we believe that the non-fluorescent fur observed here, and potentially *all* mammal fur may have some number of fluorophores present. However, only certain species can reliably be shown to fluoresce, when examined with a blacklight at 395 nm. See line 333 for possible explanations proposed by Toussaint et al 2021.**

*34. Page 10
- very clearly written!
- when you say that the fur does not naturally fluoresce in grey and red squirrels, do you mean at an observable level or at all? (my same point as above)*

**R34. Yes, while both gray and red squirrels may contain some potential fluorophores, when examined with a 395 nm blacklight no gray or red squirrel specimens showed any visible level of fluorescence. Unlike flying squirrels where many museum skins and wild specimens have been documented to fluoresce under these same conditions. See R22**

*35. Page 11
- good explanation of the porphyrin arguments*

**R35. Thank you.**

*36. Page 12
- my understanding is that there is very little good evidence that fluorescence actually serves a perceivable purpose in signaling. Since other readers will likely approach this paragraph with the same skepticism I do, would it be possible to either explain the evidence to one degree further of specificity or temper the claims?*

**R36. This is an accurate conclusion based on current evidence. While some studies have proposed potential hypothesis that could suggest fluorescence has some acting environmental pressure and is therefore somehow significant in an ecological context. To our knowledge, there is no evidence that fully supports a hypothesis that fluorescence does have an ecological role.**

**The following has been added to highlight that our research is but one piece in the fluorescent mammal puzzle, and more research on specific biochemical pathways and relationships between fluorescent mammals is needed to conclude if there is any functional purpose to mammalian fluorescence. See lines 352-362 and lines 381-382.**
