# Supplementary material for: Using mass spectrometry to investigate fluorescent compounds in squirrel fur
Source: PLoS One. 2022 Feb 22;17(2):e0257156. doi: 10.1371/journal.pone.0257156 (PMC8863215; doi:10.1371/journal.pone.0257156)
Supplement: Attachment — Submitted filename: Response to reviewers.docx [file pone.0257156.s006.docx]

**Editor comments:**

1. L16: Delete “We believe that”

**R1: We have removed the wording here.**

2. L23: consider->suggest

**R2: We have changed “consider” to “suggest”.**

3. L57: delete “well known to be”

**R3: Removed “well known to be”**

4. L64: delete “,”

**R4: Removed extra comma after non-aromatic or non-conjugated**

5. L65: delete “we would expect that”

**R5: Removed phrase**

6. L284: delete “,”

**R6: Removed unnecessary comma (now L285).**

7. L292: see L23 above

**R7: We have changed this to the word “suggest” (now L293).**

8. L311: This paragraph needs work. You did not find any differences between fluorescent and non-fluorescent fur. But clearly something is causing one to fluoresce and the other not to. What is it? You may not be able to answer this conclusively, but you need to provide some potential explanations, and suggestions for how to investigate them. If, as you suggest, the fluorphore responsible for fluorescence is common in squirrel fur, why does it only fluoresce in some cases? Could it be modified in some way? Or present in greater quantities? The following paragraph discusses tryptophan, but the discussion of porphyrin is unclear. How (as suggested in the abstract) do your results support a role for porphyrin in fluorescence? Are you suggesting that porphyrin is broken down or over accumulated in the fluorescent species you studied? Here might be a good place to mention the methodological weakness mentioned by the reviewer, i.e. that your methods were not ideal for detecting porphyrin.

**R8: Thank you. We have revised this paragraph as follows:**

**L313-333: We have further elaborated on some potential factors that may cause fluorescence in some species, but not others, and have discussed factors surrounding these hypotheses.**

**L340-360: We removed some information that was redundant with the new text at L312-339 and removed the information on porphyrin-based fluorescence that we believe is outside the scope of our conclusions.**

**L400-406: We have adjusted our conclusions here and provided three potential explanations for our results, including the degradation of porphyrins, and potential abnormal accumulations of porphyrins.**

**L414-418: We have added that fluorescent vs non-fluorescent species may also be explained by species-specific physiology supported by the findings of Pine et al. (1985).**

9. Could you not detect differences in abundance of compounds? If so, you should also mention that here

**R9: L169-170 we have added a statement to the methods to clarify that our mass spectrometer analysis can confirm presence of compounds.**
