# Supplementary material for: Using mass spectrometry to investigate fluorescent compounds in squirrel fur
Source: PLoS One. 2022 Feb 22;17(2):e0257156. doi: 10.1371/journal.pone.0257156 (PMC8863215; doi:10.1371/journal.pone.0257156)
Supplement: Attachment — Submitted filename: Hughes et al Reviewer Comments.pdf [file pone.0257156.s004.pdf]

Recommendation: Reject

This manuscript by Hughes *et al.* describes the use of Fourier transform-ion cyclotron resonance mass spectrometry (FT-ICR MS) to identify mass features present in the fluorescence pelage of either the Southern or Northern flying squirrels, but not in the non-fluorescent fur of gray squirrels, red squirrels, or eastern chipmunks. By comparing the spectrometric profiles of fluorescent vs non-fluorescent fur, the authors identified 22 tentative compounds exclusive to at least one of the fluorescent samples; however, none of the compounds were present in both flying squirrel species. Moreover, through fluorescence spectroscopy, the authors propose that both the fluorescent squirrels and some of the non-fluorescent animals exhibit fluorescence. These findings lead the authors to claim that compounds responsible for fluorescence in flying squirrels may also be present in non-fluorescent mammal fur, and that there is a currently unexplained factor that leads to excitation of these ubiquitous fluorophores in flying squirrel fur.

There are two experiments conducted in this study: fluorescence analysis and mass spectrometry analysis. The fluorescence analysis is performed by exciting extracted samples at fixed wavelength of 350 nm and scanning emissions from 400 nm to 600 nm; no rationale is given for the choice of these parameters. A glaring weakness of the selected excitation and emission wavelengths is that these parameters are ill-suited to detect the presence of porphyrins: the compounds the authors state are "...consistent with [their] findings." Most porphyrins associated with fluorescence in animals absorb strongest near 400-410 nm and emit between 600-750 nm. As such, it would be difficult to deduce porphyrin-based fluorescence from the parameters used. Moreover, the authors note that both fluorescence fur and some non-fluorescent fur show some emission activity, which leads to the unsubstantiated claim that "some factor currently unexplained likely leads to excitation of fluorophores in flying squirrel fur." The authors make no attempt to prescribe a structure to this seemingly ubiquitous fluorophore, but to this reviewer, all of the fluorescence spectra look like melanins, which are expected to be present in all types of fur regardless of fluorescence.

The second experiment executed in this study is mass spectrometry. The authors run all of the fur samples under both positive and negative mode and use commercial software to delineate features from the fluorescence samples (ventral and dorsal) from non-fluorescent samples. The authors arrive at 22 features that have some association with fluorescence but make no attempt to proffer reasonable chemical structures for these features. The authors do provide software/database generated chemical formulas for the 22 features; however, many of these formulas are blatantly unreasonable (mammals do not make compounds with formulas such as $C_9H_7F_3N_2$, $C_9H_8F_3N_3$, $C_6H_{11}I$, CH_5B_2, etc.). While this reviewer understands that these formulas are not directly generated by the authors, some oversight is needed to prevent assertions that may offend readers with chemical backgrounds.

While this reviewer is intrigued by the study, the questionable experimental designs coupled with the lack of meaningful interpretation leads me to recommend this manuscript be rejected for publication to PLOS ONE.

General Notes:

1. As a general note, manuscripts are typically written with line numbers present so it is easier for reviewers to give feedback.

2. Page 1, Abstract "Some factor currently unexplained..."

This sentence is unclear.

3. Page 2, Paragraph 2 "As the electron transitions..."

The paper says longer wavelength of light, but it does not state what it is longer than. The photon that is emitted is of lower energy (i.e. longer wavelength) that the original excitation photon, but this is not clear as written in the text. Since this is a biology paper, rather than get into nuances about this point, one could simply say "As the electron transitions back to its ground state, excess energy is lost in the form of a photon."

4. Page 2, Paragraph 2 "Since fluorescence is not..."

Replace "non-energized state" with "ground state."

5. Page 2, Paragraph 2 "Since fluorescence is not..."

The phrase "transmit fluorescence wavelengths" is very awkward and formally incorrect. One would just say "fluoresce."

6. Page 2-3, Paragraph 2 "Other molecules that contain..."

Replace "different" with "various."

7. Page 3, Paragraph 1 "The fluorescence of aromatics..."

This is incorrect. The LUMO is higher in energy than the HOMO, therefore you cannot have a de-excitation from the HOMO to the LUMO; its LUMO to HOMO. Please correct this sentence.

8. Page 3, Paragraph 1 "Aromatic rings with highly..."

The sentence is structured such that it implies the HOMO-LUMO gap emits UV light, which is technically incorrect. The HOMO-LUMO gap dictates – to an extent – the energy of the photon that is emitted, and the energy of the emitted photon dictates the wavelength of light we observe.

9. Page 3, Paragraph 3 "In fish, there are..."

Add the following citation:

Park HB et al (2019) Bright green biofluorescence in sharks derives from Bromo-Kynurenine metabolism. iScience 19:1291–1336. https://doi.org/10.1016/j.isci.2019.07.019

10. Page 4, Paragraph 2 "Tryptophan is one of..."

Tryptophan is one of the three *natural/proteinogenic* aromatic amino acids. There are many aromatic amino acids.

11. Page 5, Paragraph 1 “The colour of fluorescence...”
Fluorescence is not within a compound.

12. Page 7, Paragraph 2 “Fluorescence analysis: Aliquots (2 ml)
Remove space before (and 2.

13. Page 8, Results, Paragraph 1 “Several of these compounds...”
What does this mean? What are the exact criteria being used to determine whether compounds have the necessary chemical structure to indicate capability to fluoresce?

14. Page 9, Paragraph 4 “Our analysis of the...”
The fluorescent data looks to be consistent with eumelanin. This compound is present in keratinaceous tissue and would explain why some of the non-fluorescent specimen also exhibit emission.

15. Page 11, Paragraph 2 “Our findings are consistent...”
If the authors believe porphyrins could responsible for the fluorescence in these squirrel samples, then why not use excitation and emission wavelengths that can detect porphyrin-based fluorescence? The authors excite at 350 nm and detect emissions from 400-600 nm; however, biological porphyrins typically have absorbance maxima near 400 nm and emission maxima near 650 nm. The manner in which the authors conducted the fluorescence analysis would make it very difficult identify porphyrins as a fluorescent agent in this study.

16. Page 13, Paragraph 1 “It is also possible...”
This is easily checkable via black-light, fluorescence spectrometry, absorbance spectrometry (if porphyrin-based), etc.

17. Table 3
A potential formula of CH_5B_2 is completely ludicrous, especially as the ion observed is $[M+Cl]^-$.
